# Does conduction heterogeneity determine the supervulnerable period after atrial fibrillation?

**DOI:** 10.1007/s11517-022-02679-w

**Published:** 2022-10-12

**Authors:** Annejet Heida, Willemijn F. B. van der Does, Mathijs S. van Schie, Lianne N. van Staveren, Yannick J. H. J. Taverne, Ad J. J. C. Bogers, Natasja M. S. de Groot

**Affiliations:** 1 Unit Translational Electrophysiology, Department of Cardiology, RG-619, Erasmus Medical Center, Dr. Molewaterplein 40, 3015 GD Rotterdam, the Netherlands; 2 Department of Cardiothoracic Surgery, Erasmus Medical Center, Rotterdam, the Netherlands

**Keywords:** Atrial fibrillation, Electrophysiology, Electrical cardioversion, Atrial remodeling, Conduction velocity, Conduction disorders

## Abstract

Atrial fibrillation (AF) resumes within 90 s in 27% of patients after sinus rhythm (SR) restoration. The aim of this study is to compare conduction heterogeneity during the supervulnerable period immediately after electrical cardioversion (ECV) with long-term SR in patients with AF. Epicardial mapping of both atria was performed during SR and premature atrial extrasystoles in patients in the ECV (*N* = 17, age: 73 ± 7 years) and control group (*N* = 17, age: 71 ± 6 years). Inter-electrode conduction times were used to identify areas of conduction delay (CD) (conduction times 7–11 ms) and conduction block (CB) (conduction times ≥ 12 ms). For all atrial regions, prevalences and length of longest CB and continuous CDCB lines, magnitude of conduction disorders, conduction velocity, biatrial activation time, and voltages did not differ between the ECV and control group during both SR and premature atrial extrasystoles (*p* ≥ 0.05). Hence, our data suggest that there may be no difference in biatrial conduction characteristics between the supervulnerable period after ECV and long-term SR in AF patients.

**Graphical abstract:**

The supervulnerable period after AF termination is not determined by conduction heterogeneity during SR and PACs. It is unknown to what extent intra-atrial conduction is impaired during the supervulnerable period immediately after ECV and whether different right and left atrial regions are equally affected. This high-resolution epicardial mapping study (upper left panel) of both atria shows that during SR the prevalences and length of longest CB and cCDCB lines (upper middle panel), magnitude of conduction disorders, CV and TAT (lower left panel), and voltages did not differ between the ECV and control group. Likewise, these parameters were comparable during PACs between the ECV and control group (lower left panel). †Non-normally distributed. cm/s = centimeters per second; mm = millimeter; ms = millisecond; AF = atrial fibrillation; AT = activation time; BB = Bachmann’s bundle; cCDCB = continuous lines of conduction delay and block; CB = conduction block; CD = conduction delay; CT = conduction time; CV = conduction velocity; ECV = electrical cardioversion; LA = left atrium; LAT = local activation times; PAC = premature atrial complexes; PVA = pulmonary vein area; RA = right atrium; SR = sinus rhythm; TAT = total activation time.

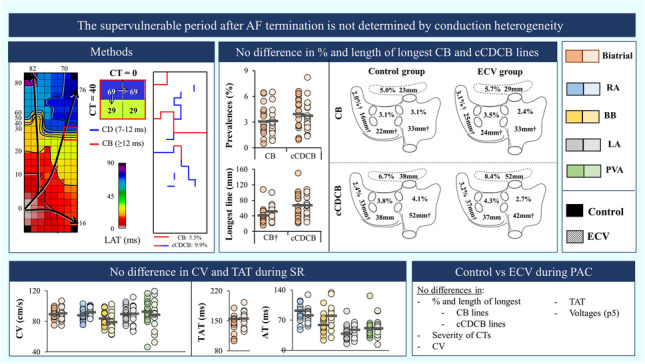

**Supplementary Information:**

The online version contains supplementary material available at 10.1007/s11517-022-02679-w.

## Introduction

The recurrence rate of atrial fibrillation (AF) after electrical cardioversion (ECV) is as high as 57% during the first month after cardioversion, with a peak incidence during the first 5 days [1]. In fact, AF even resumes within 1 or 2 min in up to 27% of patients after restoration of sinus rhythm (SR) [2–5]. This immediate recurrence of AF (IRAF) can be explained by either a high frequency of ectopic beats or the presence of a supervulnerable period immediately after ECV. Duytschaever et al. studied electrophysiological properties in a goat model after spontaneous termination of at least 5 min of AF-induced electrical remodeling and found during SR a transient shortening of the atrial effective refractory period, reduction of intra-atrial conduction velocity (CV), and shortening of the atrial wavelength compared to baseline [2]. During this so-called supervulnerable period, the atria are more susceptible to re-initiation of AF triggered by premature beats [2]. However, heterogeneity in conduction as a result of AF-induced electrical remodeling during this period during SR and premature atrial complexes has never been examined in humans. It is unknown to what extent intra-atrial conduction is impaired during this phase and whether different right and left atrial regions are equally affected. The aim of this case–control study is therefore to compare conduction heterogeneity assessed during the supervulnerable period with long-term SR at a high resolution scale. To our knowledge, this is the first study investigating differences in prevalence and severity of conduction disorders at the epicardial surface of the right atrium, Bachmann’s bundle, and left atrium including the pulmonary vein area immediately after ECV.

## Methods

### Study population and setting

The study population consisted of participants undergoing elective open-heart surgery in the Erasmus Medical Center. Indications for elective cardiac surgery were either coronary artery disease, aortic valve disease or mitral valve disease, or the combination of these. The case group consisted of AF patients who presented with AF at the onset of the surgical procedure and were electrically cardioverted to SR (structural and electrically remodeled atria). The control group consisted of AF patients who presented with SR (solely structurally remodeled atria as they were in SR for a longer period of time) [6–10]. Thus, only AF-induced electrical remodeling is studied. Participants were matched based on age [11], body mass index [12], and left atrial enlargement [13], known confounders of intra-atrial conduction disorders. In a previous paper of our group [14], we studied the impact of underlying heart disease on conduction heterogeneity during sinus rhythm and did not find any differences between patients with valvular heart disease and ischemic heart disease. Echocardiographic images were used to assess atrial dilatation. This study is approved by the institutional Medical Ethical Committee (resp. MEC 2010–054 [15] and MEC 2014–393 [16]). Prior to the surgical procedure, written informed consent was obtained from all patients. The study complied with the Declaration of Helsinki. Clinical data was extracted from electronic patient files.

### Mapping procedure

**Fig. 1:**
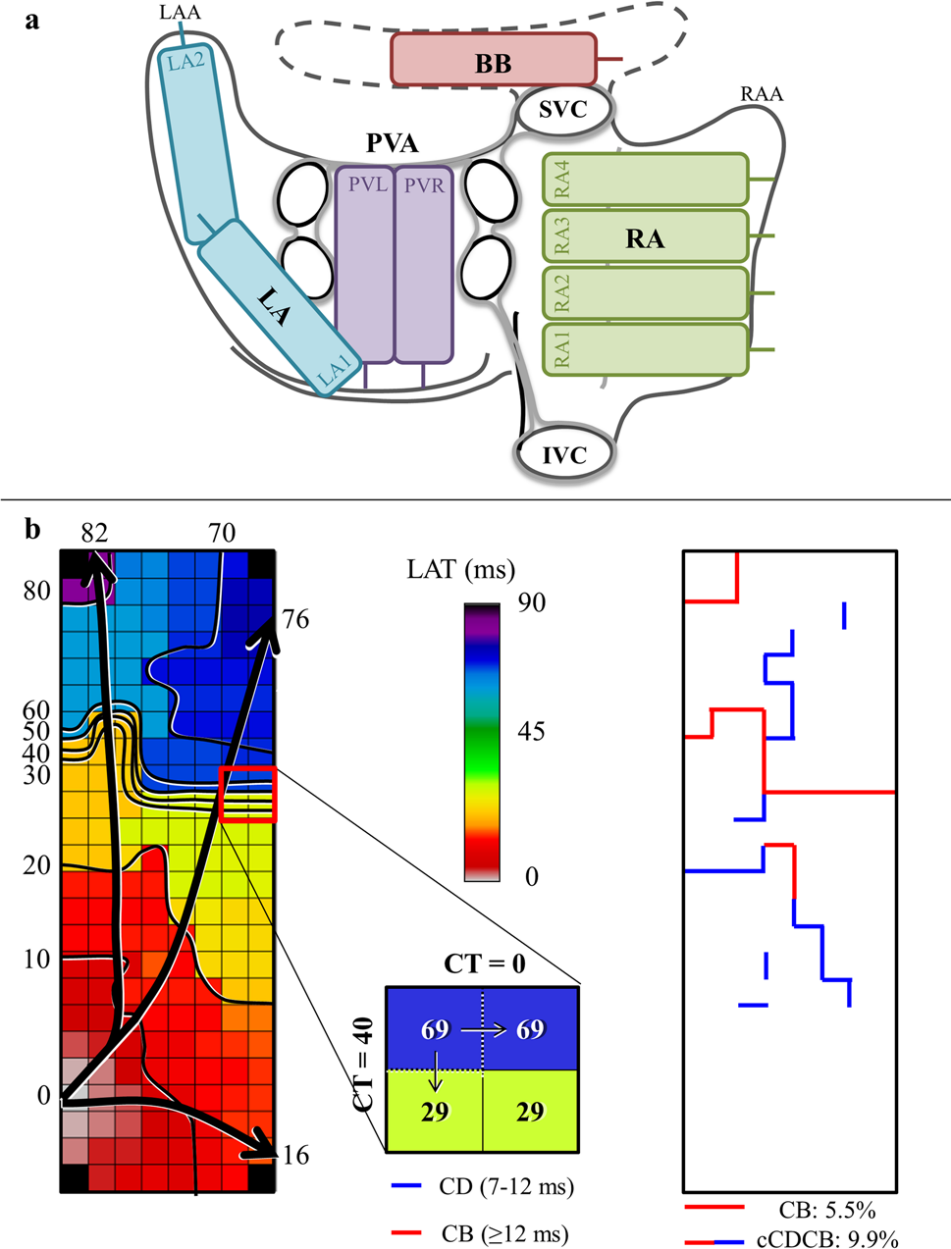
Epicardial mapping method. **a** Mapping scheme of RA (RA_1_-RA_4_), BB, LA (LA_1_-LA_2_), and PVA (PVR and PVL). **b** An example of a color-coded activation map with isochrones (black lines) drawn at 10 ms. The black arrows indicate the main wavefront directions. An example of calculation of CTs by subtracting the LAT of adjacent electrodes is shown next to the activation map. **b** An example of the corresponding CB and cCDCB map. ms, milliseconds; BB, Bachmann’s bundle; cCDCB, continuous lines of conduction delay and block lines; CB, conduction block; CD, conduction delay; CT, conduction time; IVC, inferior vena cava; LA, left atrium; LAA, left atrial appendage; LAT, local activation time; PVA, pulmonary vein area; PVL, left pulmonary vein; PVR, right pulmonary vein; RA, right atrium; RAA, right atrial appendage; SVC, superior vena cava

High-resolution epicardial mapping was performed during open-heart surgery, prior to extracorporeal circulation [17]. A pacemaker wire temporarily attached to the right atrial free wall functioned as a bipolar reference electrode. A steel wire fixed to the subcutaneous tissue of the thoracic wall was used as an indifferent electrode. Epicardial mapping was performed by shifting an unipolar 128- or a 192-electrode array (electrode diameter respectively 0.65 and 0.45 mm, inter-electrode distances of 2 mm) in a systematic order along predefined sites covering the epicardial surface of both atria (Fig. 1a), including right atrium (from the inferior caval vein up to the right atrial appendage, perpendicular to the caval veins), pulmonary vein area (from the sinus transversus, alongside the borders of the pulmonary veins towards the atrioventricular groove), left atrium (from the lower border of the left pulmonary vein along the left atrioventricular groove towards the left atrial appendage), and Bachmann’s bundle (from the tip of left atrial appendage behind the aorta towards the superior cavo-atrial junction).

At each site, 5 s of SR mapping were recorded, including unipolar epicardial electrograms, a surface electrocardiogram, a bipolar reference electrogram, and a calibration signal (amplitude: 2 mV, duration: 1000 ms). Recordings were sampled with a rate of 1 kHz, amplified (gain: 1000), filtered (bandwidth: 0.5–400 Hz), analog-to-digital-converted (16-bits), and stored on hard disk.

### Mapping data processing

The steepest negative slopes of all atrial potentials were automatically annotated with custom-made software. For each electrode, the local activation time was determined, and color-coded activation maps were reconstructed as illustrated in Fig. 1b [18, 19]. All annotations were visually verified. Mapping sites with less than 50% annotation were excluded from analysis.

### Analysis of intra-atrial conduction disorders

As previously described in a number of mapping studies, inter-electrode conduction times (CTs) were calculated by subtracting the local activation times of each electrode from the adjacent right and lower electrode (Fig. 1b) [18, 19]. Conduction delay (CD) and conduction block (CB) were defined as conduction times of respectively 7–11 ms and ≥ 12 ms, which corresponds to effective conduction velocities of respectively 17 to 29 cm/s and < 17 cm/s [20, 21]. Lines of CB and continuous CDCB (cCDCB) were defined as uninterrupted series of respectively inter-electrode CB or a combination of CD and CB (Fig. 1b). Prevalence of lines of CB and cCDCB lines are expressed as a percentage of the total available number of inter-electrode connections. In all patients, lengths of the longest CB or cCDCB line were assessed at every atrial region. The magnitude of conduction times was defined as the size of inter-electrode time differences in milliseconds and the percentage of patients with conduction times above different magnitudes was calculated. The magnitude of conduction times was analyzed in 10-ms increments. Local CV was computed as an average of velocity estimations between neighboring electrodes (longitudinal, transversal, and diagonal) using discrete velocity vectors as previously described by van Schie et al. [22]. From these local CVs, median CV and variation in CV (Δ P_5_-P_95_) were calculated for every mapping site. Total activation time and the activation time for each mapping site separately were determined by relating the first and last activation to the reference electrode. Voltage was defined as the peak-to-peak amplitude of the steepest deflection of the unipolar potential. We determined the 5th percentile of the relative frequency histograms of the voltages of all unipolar potentials and compared them between the ECV and control group. Areas of simultaneous activation were excluded from analysis in order to avoid inclusion of far field potentials.

### Intra-atrial conduction disorders during premature beats

**Fig. 2:**
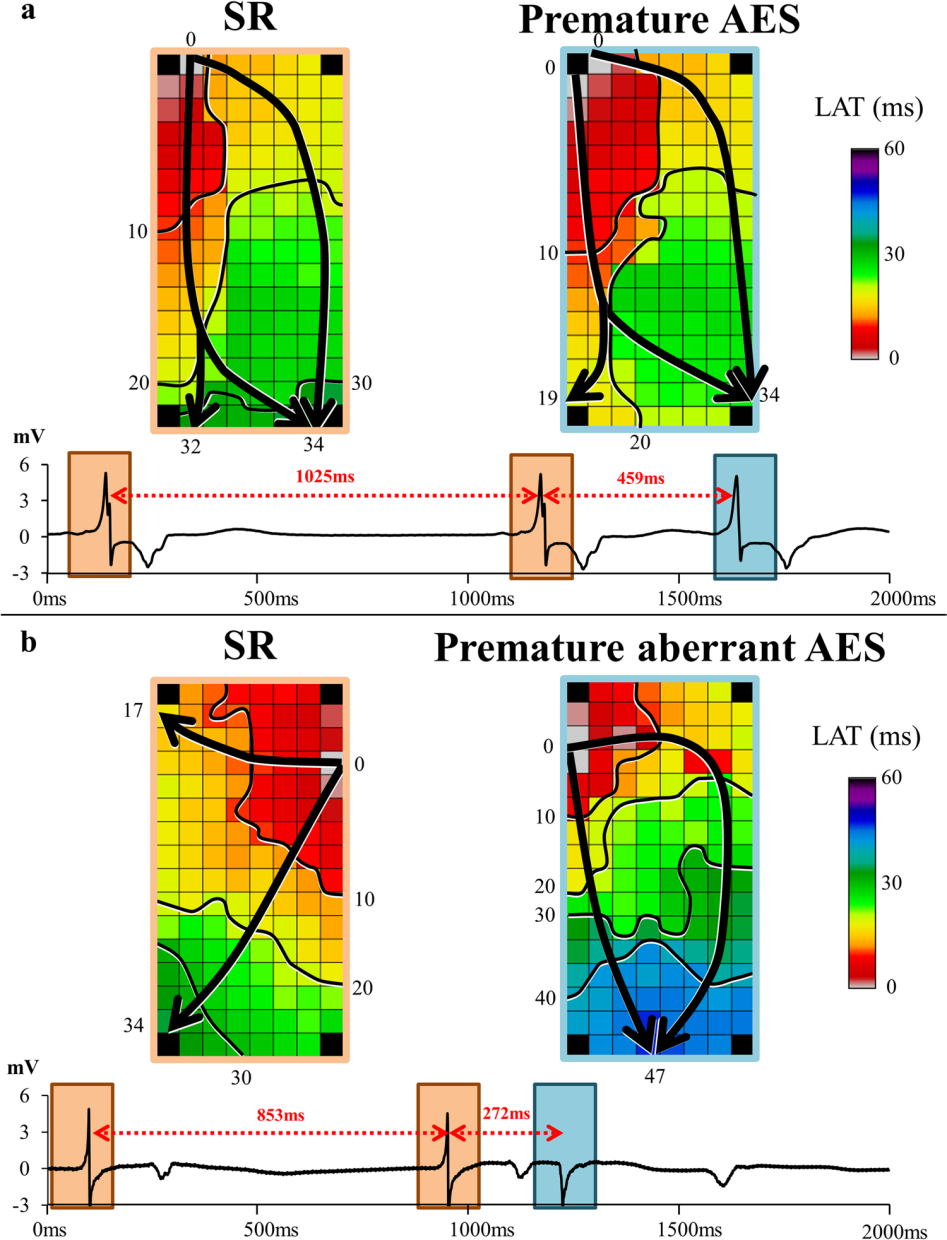
Premature atrial extrasystoles. **a** Examples of color-coded activation maps during SR (left) and during a premature atrial extrasystole (right) with a shortening in cycle length ≥ 25% compared to the previous SR beat. **b** Examples of color-coded activation maps during SR (left) and during a prematurely aberrant atrial extrasystole (right) with a shortening in cycle length ≥ 25% and a different direction of propagation compared to the previous SR beat. Isochrones (black lines) drawn at 10-ms increments. The black arrows indicate the main trajectories of activation. ms, milliseconds; mV, millivolts; AES, atrial extrasystoles; LAT, local activation time; SR, sinus rhythm

To study whether conduction disorders are more pronounced at shorter coupling intervals during the supervulnerable period, conduction heterogeneity during spontaneously occurring premature atrial complexes (PACs) was also investigated. PACs included premature and premature aberrant atrial extrasystoles (Fig. 2). PACs are defined as beats with a shortening in cycle length of ≥ 25% compared to the previous SR beat (Fig. 2a, b). Additionally, the premature aberrant beat has a different direction of propagation compared to the previous SR beat (Fig. 2b) [23]. Prematurity index of PACs was expressed as the ratio between the coupling interval of the PAC and the preceding SR cycle length:

$${\mathrm I}_{\mathrm{prematurity}}=-\frac{{\mathrm{CL}}_{\mathrm{PAC}}}{{\mathrm{CL}}_{\mathrm{SR}}}\bullet100\%$$

${\mathrm{CL}}_{\mathrm{PAC}}$ equals the cycle length of the spontaneous PAC and ${\mathrm{CL}}_{\mathrm{SR}}$ the cycle length of the preceding two sinus beats. The difference (Δ) of conduction parameters between the previous SR beat and the PACs were compared between the ECV and the control group.

### Statistical analysis

Statistical analysis was performed with SPSS version 25 (IBM Corporation, Armonk, NY). All data were tested for normality using Shapiro–Wilk test. Normally distributed continuous data were expressed as mean ± standard deviation and skewed data as median (interquartile range). A paired samples *t*-test or Wilcoxon signed rank test was used to compare continuous parameters for the comparison of SR between the ECV and the control group. For the comparison of continuous data during PACs, an independent samples *t*-test or Mann–Whitney *U* test was performed. Categorical data are expressed as absolute numbers (percentages) and analyzed with (McNemar’s symmetry) χ^2^ or McNemar’s exact test if appropriate. For the comparison of magnitude of conduction times between the ECV and control group, correction for multiple testing was applied. Corrected *p* values will be reported. A two-sided *p* value of < 0.05 was considered statistically significant.

## Results

### Study population

As presented in Table 1, baseline characteristics between the ECV (*N* = 17, 73 ± 7 years; 11 (64.7%) male) and control group (*N* = 17, 71 ± 6 years; 12 (70.6%) male) did not differ (all *p* ≥ 0.05). Participants in the ECV group had either paroxysmal AF (*N* = 6, 35.3%), persistent AF (*N* = 9, 52.9%), or longstanding persistent AF (*N* = 2, 11.8%), while in the control group participants had paroxysmal AF (*N* = 11, 64.7%) or persistent AF (*N* = 6, 35.3%) (*p* = 1.00). Patients in the ECV group had an AF episode duration of 1 month (0.5–4.0) before AF was terminated. Patients in the control group were 54 (13–234) days in SR before surgery.

**Table 1:** Characteristics of participants

	ECV group (*N* = 17)	Control group (*N* = 17)	*p* value
Age—years (mean ± SD)	73 ± 7	71 ± 6	0.32
Male sex—*N* (%)	11 (64.7)	12 (70.6)	1.00
BMI—kg/m^2^ (mean ± SD)	27 ± 5	26 ± 5	0.42
History of AF—*N* (%)	17 (100.0)	17 (100.0)	1.00
Paroxysmal	6 (35.3)	11 (64.7)	*p* ≥ 0.017*
Persistent	9 (52.9)	6 (35.3)	*p* ≥ 0.017*
Longstanding persistent	2 (11.8)	0 (0.0)	*p* ≥ 0.017*
Underlying heart disease—*N* (%)			1.00
IHD	2 (11.8)	2 (11.8)	
(i)VHD	15 (88.2)	15 (88.2)	
AVD	2 (11.8)	4 (23.5)	
AVD and CAD	2 (11.8)	1 (5.9)	
MVD	9 (52.9)	8 (47.1)	
MVD and CAD	2 (11.8)	2 (11.8)	
Echocardiography			
LVF—*N* (%)			0.13
Normal	11 (64.7)	16 (94.1)	
Mild dysfunction	3 (17.6)	0 (0.0)	
Moderate dysfunction	3 (17.6)	0 (0.0)	
Severe dysfunction	0 (0.0)	1 (5.9)	
LAVI—ml/m^2^ (median (IQR))	47 (43–63)†	46 (38–66)‡	0.72
Medication—*N* (%)			
Antiarrhythmic drugs			0.45
Class I	0 (0.0)	0 (0.0)	
Class II	11 (64.7)	8 (47.1)	
Class III	4 (23.5)	4 (23.5)	
Class IV	1 (5.9)	1 (5.9)	
Digoxin	5 (29.4)	3 (17.6)	0.50

*N*, number; *SD*, standard deviation; *AF*, atrial fibrillation; *AVD*, aortic valve disease; *BMI*, body mass index; *CAD*, coronary artery disease; *ECV*, electrical cardioversion; *IHD*, ischemic heart disease; *LA*, left atrium; *LAVI*, left atrial volume index; *LVF*, left ventricular function; *MVD*, mitral valve disease; *(i)VHD*, (ischemic and) valvular heart disease. †*N* = 15. ‡*N* = 12. *Bonferroni correction was applied

### Mapping data characteristics

In the ECV and control group, a total of respectively 164,099 unipolar potentials (9192 potentials/patient (7421–11,250)) and 149,521 unipolar potentials (8418 potentials/patient (7112–10,831)) were available for further analysis (*p* = 0.52). Due to simultaneous activation, 2.3% of the potentials in the ECV group and 1.1% of the potentials in the control group were excluded from analysis. SR cycle length during epicardial mapping was 788 ms (736–894) in the ECV group and 855 ms (764–962) in the control group (*p* = 0.10).

### Biatrial conduction

**Fig. 3:**
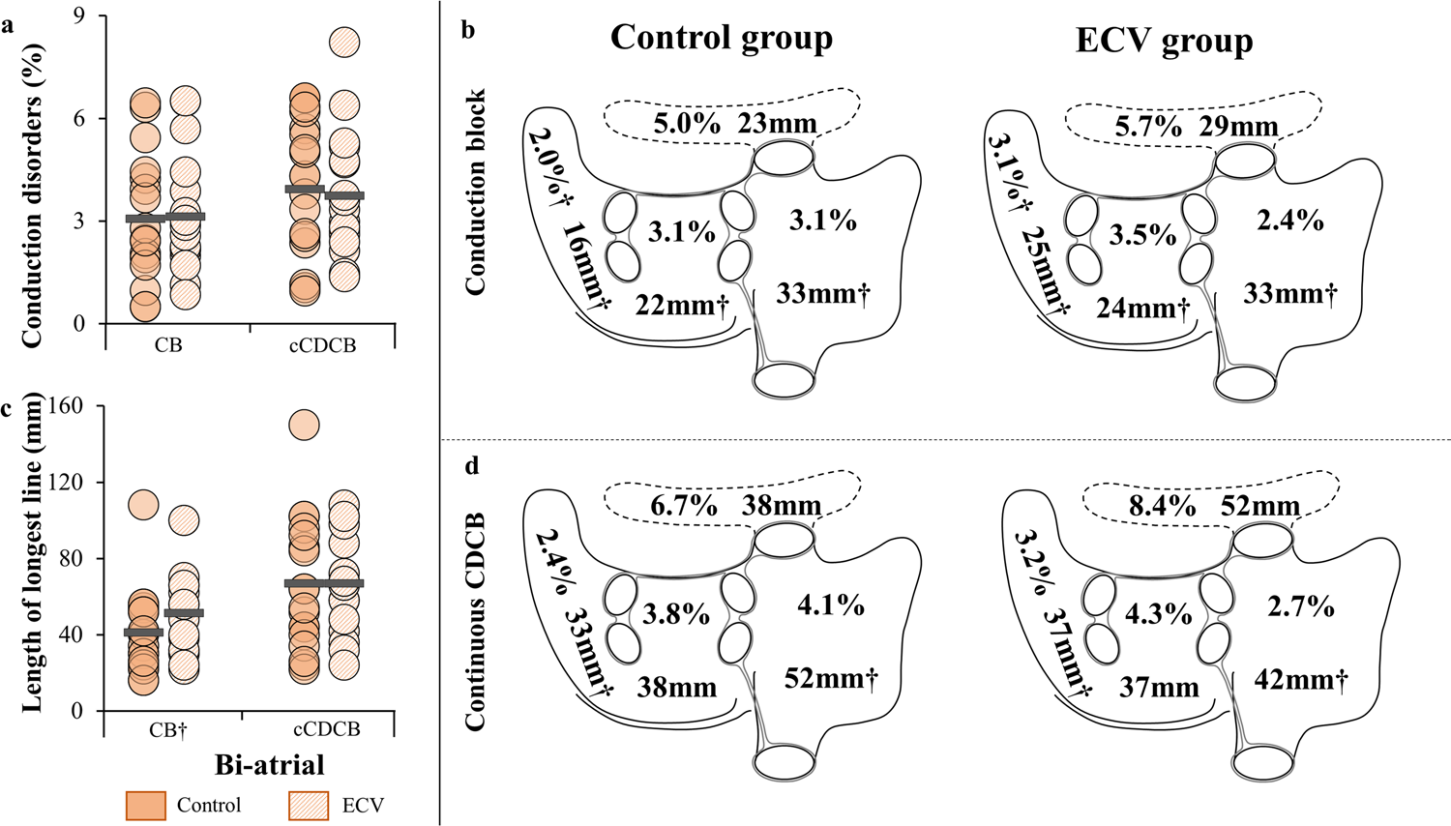
Prevalences and length of longest CB and cCDCB lines. **a** Prevalence of CB and cCDCB in both atria. **b** Spatial distribution of prevalences of CB and length of longest CB lines. **c** Length of longest lines of CB and cCDCB in both atria. **d** Spatial distribution of prevalences of cCDCB and length of longest cCDCB lines. †Non-normally distributed. mm, millimeter; ms, milliseconds; cCDCB, continuous lines of conduction delay and block; CB, conduction block; ECV, electrical cardioversion

In the entire study population, each patient in the ECV group, as well as in the control group, had areas of CD and CB. Differences in prevalences and length of longest lines of CB and cCDCB in both atria between the ECV and control group are shown in Fig. 3a, c. As illustrated in Fig. 3a, the prevalence of CB and cCDCB in both atria did not differ between the control and ECV group (CB: 3.1 ± 1.7% vs. 3.1 ± 1.9%, *p* = 0.93; cCDCB: 3.7 ± 1.8% vs. 3.9 ± 1.9%, *p* = 0.78). Additionally, the length of the longest lines of both CB and cCDCB was the same in patients immediately after ECV and during long-term SR (CB: 48 mm (31–66) vs. 40 mm (25–53), *p* = 0.23; cCDCB: 67 ± 26 mm vs. 67 ± 35 mm, *p* = 1.00) (Fig. 3c).

Regional differences in prevalences and the length of longest lines of CB and cCDCB between the control and the ECV group are shown in the Fig. 3b, d and Supplemental Table 1. Conduction disorders are observed in both groups at all locations, but mainly at Bachmann’s bundle. Figure 3b, d and Supplemental Table 1 show that both the prevalence of CB and cCDCB as well as the length of the longest CB lines and cCDCB lines at every location did not differ between the ECV and control group (all *p* ≥ 0.05).

**Fig. 4:**
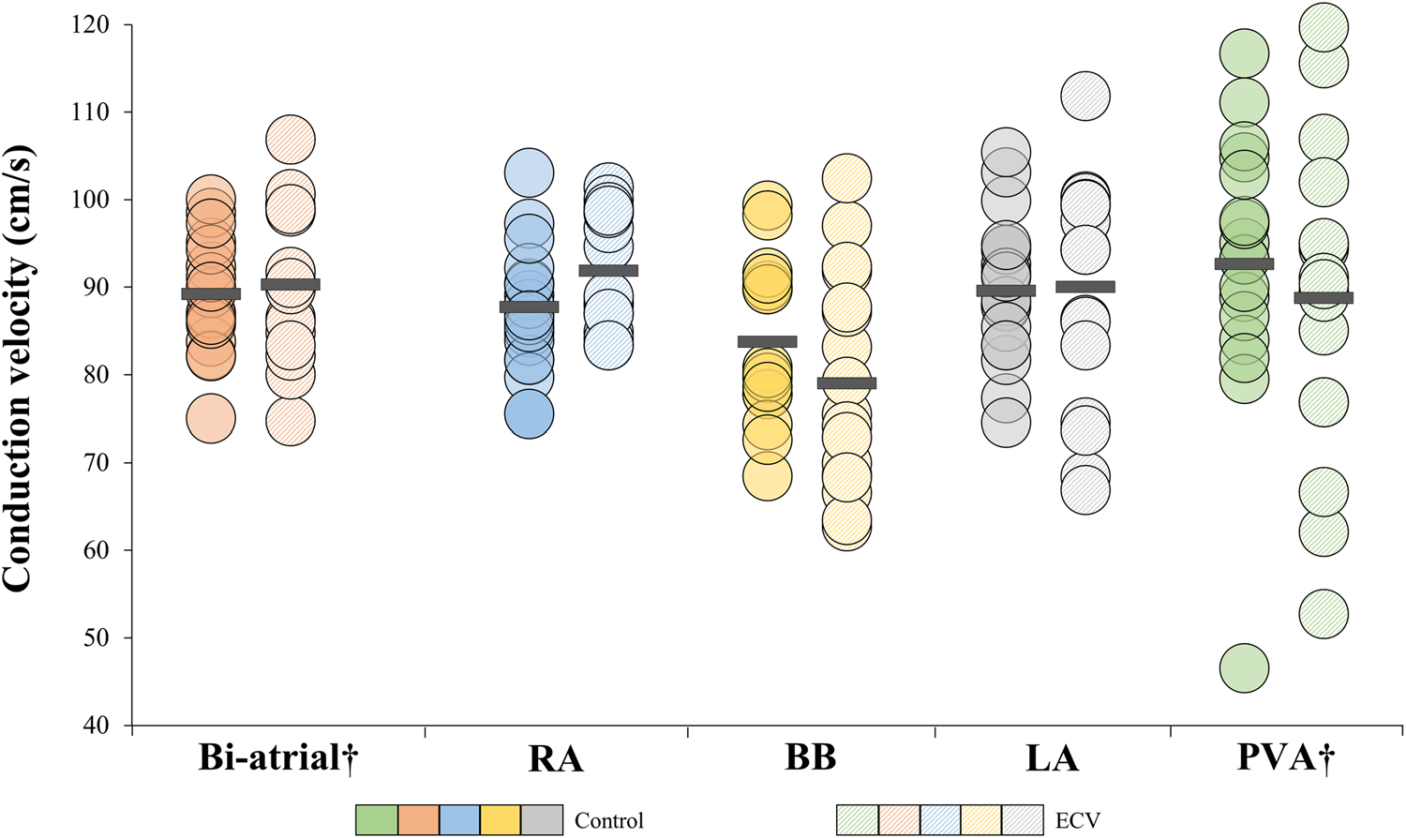
Biatrial and regional conduction velocity. Left panel: biatrial median conduction velocity displayed for each patient. Right panel: median conduction velocity displayed for each patient per region separately. †Non-normally distributed. cm/s, centimeter per second; BB, Bachmann’s bundle; ECV, electrical cardioversion; LA, left atrium; PVA, pulmonary vein area; RA, right atrium

Figure 4 shows the median CV for each patient in both atria and for each location separately. Biatrial median CV was not reduced in the ECV group (90 cm/s (84–99) vs. 89 cm/s (85–95), *p* = 0.69). Biatrial variation in CV also did not differ between both groups (Δ P_5_-P_95_: 127 cm/s (123–132) vs. 125 cm/s (121–136), *p* = 0.87). Comparing CV for each location separately, again no differences in median CV were found between the ECV and the control group (right atrium: 92 ± 7 cm/s vs. 88 ± 7 cm/s, *p* = 0.11; Bachmann’s bundle: 80 ± 12 cm/s vs. 84 ± 9 cm/s, *p* = 0.22; pulmonary vein area: 90 cm/s (77–98) vs. 93 cm/s (85–104), *p* = 0.34; left atrium: 90 ± 13 cm/s vs. 90 ± 8 cm/s, *p* = 0.90). As shown in Supplemental Table 1, the variation in CV per location also was comparable between patients in the ECV and control group (all *p* ≥ 0.05).

### Severity of conduction disorders

**Fig. 5:**
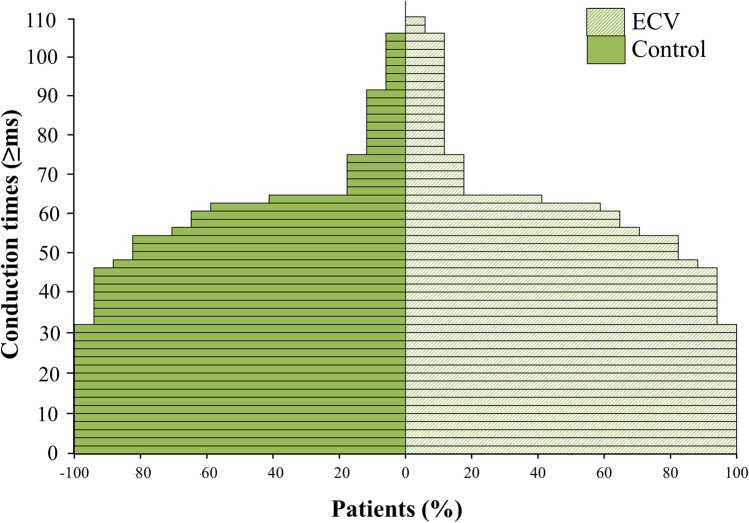
Severity of conduction disorders. Magnitude of CTs measured after ECV and during long-term SR for the entire study population in increments of 10 ms. ms, milliseconds; CTs, conduction times; ECV, electrical cardioversion; SR, sinus rhythm

Figure 5 shows the magnitude of conduction times in both atria for the ECV and control group separately. Each patient in both groups had at least one CT ≥ 32 ms. The magnitude of conduction times was comparable in the ECV and control group (Bonferroni corrected *p* ≥ 0.006). By comparing the different atrial regions separately between both groups, again there were no differences in the magnitude of conduction times (right atrium: Bonferroni corrected *p* ≥ 0.008; Bachmann’s bundle: Bonferroni corrected *p* ≥ 0.005; pulmonary vein area: Bonferroni corrected *p* ≥ 0.01; left atrium: Bonferroni corrected *p* ≥ 0.01).

### Relation between conduction heterogeneity and biatrial activation time

**Fig. 6:**
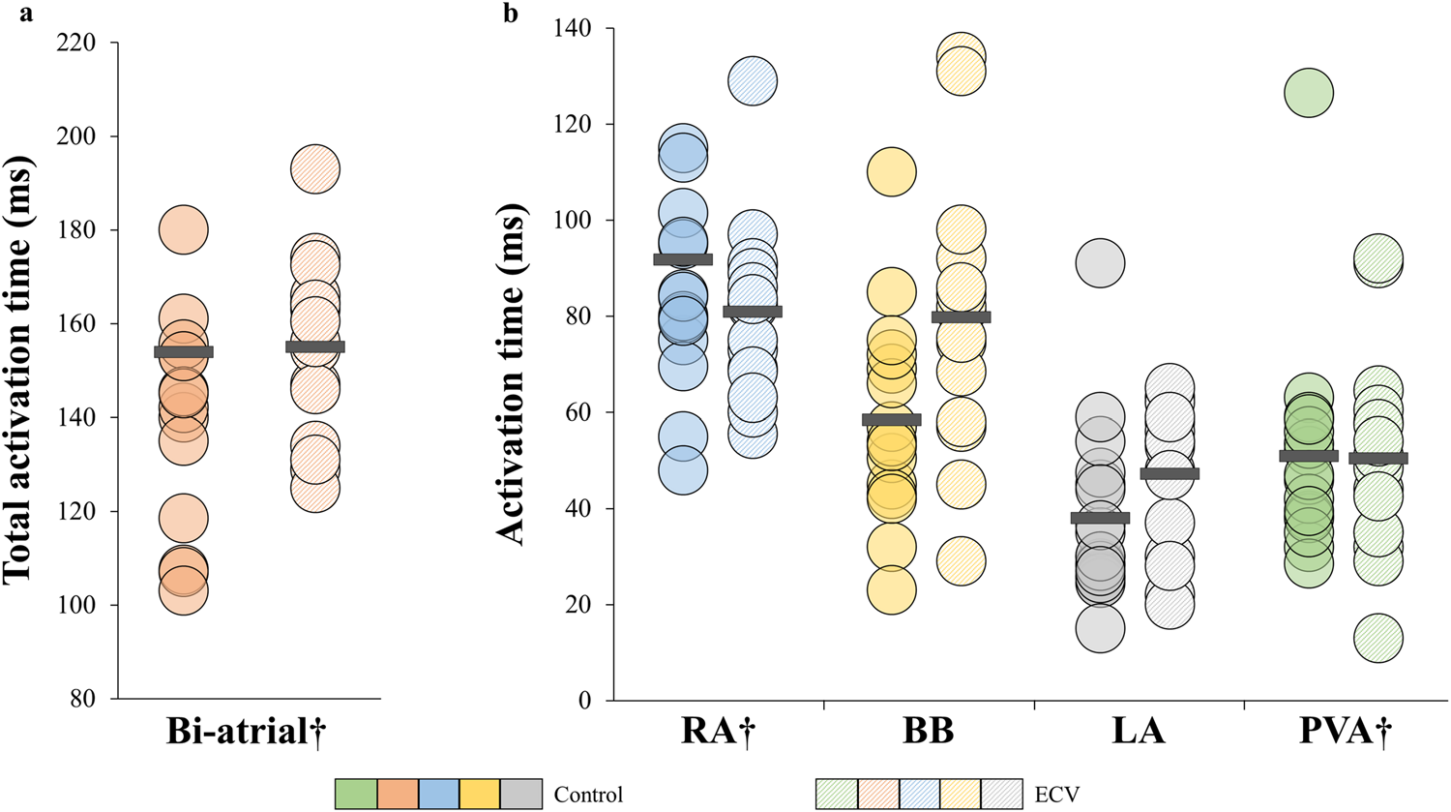
Total activation time. **a** Biatrial total activation time displayed for each individual patient. **b** Activation time displayed for each patient per region separately. †Non-normally distributed. ms, milliseconds; BB, Bachmann’s bundle; ECV, electrical cardioversion; LA, left atrium; PVA, pulmonary vein area; RA, right atrium

Figure 6 illustrates for each patient the total activation times (Fig. 6a) and the activation time per region separately (Fig. 6b). The supervulnerable period was not associated with a prolonged biatrial total activation times (158 ms (137–166) vs. 145 ms (122–160), *p* = 0.41) or a prolonged activation time for each location separately (all *p* ≥ 0.05). Activation time was longest at the right atrium (ECV: 82 ms (69–90), control: 84 ms (76–110), *p* = 0.57).

### Unipolar voltages

Comparison of the 5th percentile of all biatrial voltages between the control and ECV group did not reveal lower unipolar voltages during the supervulnerable period (Table 2; ECV group: 0.8 ± 0.4 mV vs. control group 0.9 ± 0.5 mV; *p* = 0.31). When comparing the 5th percentile of voltages for each location separately, there were also no differences between both groups (all *p* ≥ 0.05).

**Table 2:** Unipolar voltages in the ECV and control group

	ECV (*N* = 17)	Control (*N* = 17)	*p* value
Biatrial—mV (mean ± SD)	0.7 ± 0.3	0.9 ± 0.5	0.31
Right atrium—mV (median (IQR))	1.0 (0.7–1.3)	0.9 (0.5–1.3)	0.83
Bachmann’s bundle—mV (mean ± SD)	1.0 ± 0.6	1.1 ± 0.8	0.49
Pulmonary vein area—mV (median (IQR))	0.6 (0.5–1.4)	0.9 (0.6–1.2)	0.94
Left atrium—mV (mean ± SD)	1.2 ± 0.7	1.6 ± 0.7	0.22

*mV*, millivolts; *IQR*, interquartile range; *SD*, standard deviation; *ECV*, electrical cardioversion

### Conduction disorders during premature beats

Seven patients (41%) in the control group had a total of 11 PACs (4 premature atrial extrasystole (36%); 7 premature aberrant atrial extrasystole (64%)) whereas in the ECV group, seven patients (41%) had a total of 22 PACs (6 premature atrial extrasystole (27%); 16 premature aberrant atrial extrasystole (73%)). The prematurity index of the PACs did not differ between the control and ECV group (61.2 ± 10.3% versus 55.6 ± 12.6%, *p* = 0.22).

Table 3 shows for both groups the difference (Δ) in conduction disorders during the PAC compared to the corresponding SR beat. The increase in conduction disorders was not more pronounced during the supervulnerable period, as the Δ prevalence and Δ length of longest CB and cCDCB lines did not differ between both groups (all *p* ≥ 0.05). Additionally, Δ CV was similar between the control and the ECV group as the CV decreased with respectively 11 ± 13 cm/s and 6 ± 19 cm/s between SR and PACs (*p* = 0.48). The supervulnerable period was also not associated with a more pronounced decrease of the 5th percentile of the voltage histograms in patients after ECV (− 0.3 mV (− 1.0–0.4) vs. − 0.2 mV (− 2.2–0.6), *p* = 0.87).

**Table 3:** Differences (Δ) in conduction during PACs compared SR

	ECV (*N* = 22)	Control (*N* = 11)	*p* value
Δ CB			
Prevalence—% (median (IQR))	1.0 (− 0.7–3.6)	2.6 (0.0–5.6)	0.30
Length of longest CB line—mm (median (IQR))	6.0 (2.0–10)	12.5 (3.0–27.0)	0.27
Δ cCDCB			
Prevalence—% (mean ± SD)	3.6 ± 6.2	1.1 ± 5.3	0.23
Length of longest cCDCB line—mm (median (IQR))	0.0 (− 2–8)	18 (− 12.0–28)	0.13
Δ CV—cm/s (mean ± SD)	− 6 ± 19	− 11 ± 13	0.48
Δ P_5_ of unipolar voltages—mV (median (IQR))	− 0.3 (− 1.0–0.4)	− 0.2 (− 2.2–0.6)	0.87

*cm/s*, centimeters per second; *SD*, standard deviation; *IQR*, interquartile range; *CB*, conduction block; *cCDCB*, continuous lines of conduction delay and block; *CV*, conduction velocity; *PAC*, premature atrial complexes

## Discussion

### Key findings

This high-resolution intra-operative mapping study is the first to investigate biatrial heterogeneity in conduction during the so-called supervulnerable period immediately after ECV. Compared to long-term SR, no increased conduction heterogeneity was found immediately after ECV, since the frequency and severity of conduction disorders, as well as CV and TAT, did not differ during SR between the control and ECV group. Additionally, conduction disorders during PACs were not more pronounced immediately after ECV. Hence, our data suggest that the supervulnerable period may not be characterized by impaired intra-atrial conduction.

### Conduction disorders as a predictor for early atrial fibrillation recurrences

Rosenbaum introduced the term “domestication of AF,” meaning that the longer the duration of AF episodes, the more difficult it becomes to achieve SR. After termination of AF, 27% of patients have an IRAF within 1 min after successful ECV [3–5]. At a higher heart rate, e.g., during AF, atrial CV will decrease while the wavelength of the atrial impulse and the atrial effective refractory period shortens [24, 25]. These changes during AF promote re-entry as they reduce the likelihood that a wavefront circling around a line of CB collides with its refractory tail [26]. After AF termination, it is generally assumed that the combination of increased dispersion of the atrial effective refractory period and a reduced CV in combination with the presence of triggers such as PAC may increase the susceptibility to AF recurrence.

Duytschaever et al. examined the supervulnerable phase immediately after AF termination in goats with non-remodeled and electrically remodeled atria (48 h of electrically maintained AF) [2]. Baseline atrial effective refractory period, intra-atrial CV, and atrial wavelength were determined [2]. After the baseline study, AF was induced lasting at least 5 min and all measures were repeated immediately after spontaneous restoration of SR [2]. They found transient shortening of the atrial effective period, reduction of intra-atrial CV during SR, and shortening of the atrial wavelength compared to baseline [2]. These observations implied the existence of a vulnerable substrate for initiation of reentry after AF termination in goats. One possible explanation for slowing of conduction after AF termination is a decrease in sodium and increase in potassium currents due to high atrial rates during AF [27–30]. The resting membrane potential, and as a result the driving force for sodium ion exchange, will decrease resulting in a lower action potential velocity upstroke and thus a lower CV [2]. However, we did not observe a reduction of CV in humans immediately after AF termination. We found that intra-atrial conduction during the supervulnerable period and long-term SR were comparable. Also during PACs, there was no reduction of CV. In other words, our findings suggest that an increased susceptibility to AF re-initiation during the so-called supervulnerable period may be not determined by a reduction in CV.

A possible explanation may be that the normalization of intracellular sodium concentrations is restored within only a few SR beats and is not present for 1 to 2 min as previously suggested. Another explanation may be the duration of AF and its impact on electrical remodeling [31, 32]. In our ECV group, patients had 1-month AF before termination of AF ranging (IQR) between 2 weeks and 4 months. Only 2 patients had longstanding persistent AF (AF duration of 1 year and 1.5 year) before termination, while longer AF episodes are correlated with more electrical remodeling and thus a reduced CV [31, 32].

In humans, atrial conduction during the supervulnerable period has not been previously investigated. However, a few studies reported on the reversal of electrical remodeling over time after termination of AF. Yu et al. performed endocardial mapping of the left atrium and right atrium 30 min (*t* = 30) after restoration of SR in humans and studied conduction times during four consecutive days using two ten-polar electrode catheters positioned at the right atrial appendage and distal coronary sinus [33]. Conduction times were measured from the second to the fifth pairs of electrodes (5-mm inter-pair distance), while the first pair was used for pacing at a basic cycle length of 700 ms [33]. After termination of AF, inter-atrial conduction did not change during these 4 days [33]. We studied conduction times during SR as a measure of inter-electrode differences in local activation time ≥ 12 ms (CB) and as the activation time of the right atrium and left atrium. Moreover, we investigated these conduction properties during the supervulnerable period rather than the reversal of electrical remodeling over time starting at *t* = 30 min. However, our findings that there are no differences in frequency of CB and activation time of the right atrium and left atrium during the supervulnerable period are consistent with these findings. Additionally, Yu et al. [33] examined surface electrocardiograms over the same time course after AF termination using the duration of the *p* wave as a measure of total activation times in patients with persistent AF. They found no change in *p* wave duration over time [33]. In contrast, in another study, *p* wave duration was prolonged within 5 to 20 min after AF termination compared to 24 h and 1 month post-conversion [34]. However, in both studies, there were no measurements during the supervulnerable period. The control group in our study was in SR for approximately 1.5 months and still no differences in intra-atrial conduction were found between control and ECV group.

### Premature beats as a trigger for early atrial fibrillation recurrences

Triggers, e.g., PACs, play an important role in AF onset in patients with an IRAF [2, 35]. In our study population, a higher incidence of PACs was present during the supervulnerable period compared to long-term SR. In both groups, seven patients had PACs, but 22 PACs were found in the ECV group, while only 11 PACs were found in the control group. A possible mechanism that enhances PAC-initiated IRAF is the occurrence of intracellular calcium overload due to the previous high-rate AF episode promoting late phase 3 early afterdepolarization-induced PACs [36]. The high AF rates result in an increase in intracellular sodium leading to cellular calcium load mediated by sodium-calcium exchange [36]. After AF termination, strong calcium release in the sarcoplasmic reticulum stimulates extrusion of calcium through sodium-calcium exchanger [36]. As a result, a transient period of hypercontractility occurs [37]. Additionally, the inward current of calcium mediated by the exchanger is most likely responsible for the transient action potential duration prolongation and early afterdepolarizations [36].

Additionally to the occurrence of atrial triggers, IRAF requires a vulnerable substrate for reentry. In our study population, even conduction disorders caused by PACs were not more pronounced during the supervulnerable period. A limitation is that we did not study PACs that did indeed trigger an IRAF, yet the prematurity of PACs is comparable to previously reported PACs inducing AF. In the goat model of AF, all IRAF episodes were triggered by PACs with coupling intervals ranging between 310 and 580 ms; ectopic beats with a coupling interval > 600 ms never resulted in IRAF [2]. In humans, the coupling intervals of PACs initiating IRAF were shorter (418 ms) than PACs which did not initiate IRAF (661 ms) (*p* < 0.05) [3]. PACs in our study population had a mean coupling interval of 482 ms (prematurity index: 61.2%) and 470 ms (prematurity index: 55.6%) in respectively the control and ECV group. Although no IRAF was initiated, these coupling intervals were short enough to initiate AF.

### Potential other mechanisms leading to early atrial fibrillation recurrences

Since intra-atrial conduction is not impaired after AF termination during both SR and PACs, other mechanisms may be responsible for the occurrence of IRAF. As previously mentioned, an increased dispersion of atrial effective refractory period, a reduced CV, and the frequency of triggers may enhance the susceptibility to AF recurrences. In the present study, we did not investigate atrial effective refractory period, as our study was designed to study conduction during SR and PACs. However, several other human studies found a shortening of atrial effective refractory period during the supervulnerable period [34, 38–40]. Additionally, a significant dispersion of atrial refractoriness between different right atrium sites was present [38]. This, in combination with a higher frequency of PACs, may be a possible explanation for the occurrence of IRAF, as it facilitates the likelihood of encountering unidirectional conduction block, which is a prerequisite for development of re-entrant circuits.

In the present study, biatrial CV was not reduced in the ECV group (*p* = 0.69). However, we have not studied the rate-dependent slowing of CV (CV restitution) which may precede AF initiation [41]. Narayan et al. showed that patients with paroxysmal AF had steep CV restitution interacting with steep action potential restitution, which may cause rapid tachycardias to initiate AF [41, 42]. On the other hand, patients with persistent AF, with more advanced remodeling of the atria, and broad CV restitution developed AF at lower heartrates [41–44]. However, the precise mechanism underlying the relationship between CV restitution and AF initiation is still unclear.

### Low voltage areas during the supervulnerable period

In our present study, we found no relationship between low voltage areas and the supervulnerable period. Little is known about the impact of electrical remodeling on unipolar voltages. As previously mentioned, it is likely that due to electrical remodeling during AF, sodium current is reduced resulting in a decrease of voltages displayed in the unipolar electrogram which may still be present after AF termination [27–30]. However, we did not find low voltage areas during the supervulnerable period.

### Study limitations

Patients with a history of AF may have had variable degrees of atrial remodeling as some patients had persistent or longstanding persistent AF, while other patients had paroxysmal AF. Even if we perform a subanalysis in patients with paroxysmal AF between the ECV and control group, there were no differences found in any of the conduction parameters (see Supplemental Table 2). In patients with persistent and longstanding persistent AF, comparable results were found (see supplemental Table 3). Additionally, PACs triggering AF were not investigated.

### Clinical relevance

This study is the first to investigate conduction disorders due to AF-related electrical remodeling immediately after ECV in high resolution of the entire atrial surface. Since intra-atrial conduction is not impaired after AF termination during both SR and PACs compared to long-term SR, it is suggested that IRAF is not enhanced by conduction disorders. To further investigate conduction impairment during the shortest coupling intervals, programmed electrical stimulation reaching atrial refractoriness should be performed to examine CV restitution in relation to AF initiation. Other mechanisms, such as an increased dispersion of atrial effective refractory period and the frequency of triggers, may also be possible explanations for the occurrence of IRAF. These findings help to better understand the mechanism behind the IRAF and improve treatment strategies aimed at eliminating IRAF.

### Conclusion

This high-resolution intra-operative mapping study is the first to investigate characteristics of biatrial conduction immediately after ECV during the so-called supervulnerable period. Compared to long-term SR, there was no impaired intra-atrial conduction immediately after ECV. These observations suggest that the supervulnerable period is not characterized by increased conduction heterogeneity during SR or PACs. However, to further investigate conduction impairment during the shortest coupling intervals, programmed electrical stimulation reaching atrial refractoriness should be performed to examine CV restitution.

## Supplementary Information

Below is the link to the electronic supplementary material.

Supplementary file1 (DOCX 22 KB)
