# Supplementary material for: Does conduction heterogeneity determine the supervulnerable period after atrial fibrillation?
Source: Med Biol Eng Comput. 2022 Oct 12;61(4):897–908. doi: 10.1007/s11517-022-02679-w (PMC9988743; doi:10.1007/s11517-022-02679-w)
Supplement: Supplementary file 1 — Supplementary file1 (DOCX 22 KB) [file 11517_2022_2679_MOESM1_ESM.docx]

**Supplemental Table 1.**

|  | **ECV**  **(N=17)** | **Control**  **(N=17)** | **P Value** |
| --- | --- | --- | --- |
| **Prevalence of CB**  **RA – % (mean ± SD)**  **BB – % (mean ± SD)**  **PVA – % (mean ± SD)**  **LA** **– % (median (IQR))**  **Length of maximum lines of CB**  **RA – mm (median (IQR))**  **BB – mm (mean ± SD)**  **PVA – mm (median (IQR))**  **LA** **– mm (median (IQR))**  **Prevalence of cCDCB**  **RA – % (mean ± SD)**  **BB – % (mean ± SD)**  **PVA – % (mean ± SD)**  **LA** **– % (median (IQR))**  **Length of maximum lines of cCDCB**  **RA – mm (median (IQR))**  **BB – mm (mean ± SD)**  **PVA – mm (mean** ± **SD)**  **LA** **– mm (median (IQR))**  **Variation of CV (Δ P5-P95)**  **RA – cm/s (mean ± SD)**  **BB –cm/s (mean ± SD)**  **PVA – cm/s (mean ± SD)**  **LA – cm/s (mean ± SD)** | 2.4 ± 1.3  5.7 ± 4.0  3.5 **±**  4.4  1.9 (0.7 – 4.7)  30 (20 – 45)  29 ± 15  14 (7 – 23)  18 (8 – 30)  2.7 ± 1.6  8.4 ± 5.0  4.3 ± 4.3  3.2 ± 2.8  36 (25 – 64)  52 ± 25  37 ± 28  30 (18 – 49)  118 ± 9  125 ± 12  129 ± 12  139 ± 11 | 3.1 ± 2.5  5.0 ± 3.6  3.1 **±** 3.3  1.5 (0.4 – 3.9)  26 (11 – 48)  23 ± 12  15 (11 – 32)  32 (20 – 41)  4.1 ± 3.0  6.7 ± 5.3  3.8 ± 3.9  2.4 ± 2.0  38 (25 – 90)  38 ± 20  38 ± 22  32 (20 – 41)]  117 ± 12  130 ± 13  132 ± 13  137 ± 9 | 0.31  0.93  0.79  0.52  0.11  0.36  0.66  0.96  0.11  0.53  0.77  0.40  0.86  0.19  0.88  0.96  0.92  0.20  0.51  0.58 |

**IQR** = interquartile range; **SD** = standard deviation; **cCDCB** = continuous lines of conduction delay and conduction block; **CB**  = conduction block; **CV**  = conduction velocity; **ECV** = electrical cardioversion; **BB** = Bachmann’s bundle; **LA**  = left atrium; **PVA** = pulmonary vein area; **RA** = right atrium.

**Supplemental Table 2.**

|  | **ECV**  **(N=6)** | **Control**  **(N=11)** | **p value** |
| --- | --- | --- | --- |
| **CB**  **Prevalence – % (mean±SD )**  **Length of longest CB line – mm ( median (IQR))** | 3.1±1.9  48 (30 – 67) | 3.0±1.8  30 (24 – 56) | 0.92  0.35 |
| **cCDCB**  **Prevalence - % (mean±SD)**  **Length of longest cCDCB line – mm (mean±SD)** | 3.8±1.8  68±30 | 3.8±2.0  70±41 | 0.96  0.90 |
| **CV – cm/s (mean±SD)** | 79±7 | 91±6 | 0.09 |
| **Variation of CV (Δ P5-P95)** | 126±6 | 129±10 | 0.56 |
| **P_5_ of unipolar voltages – mV (median (IQR))** | 1.0±0.4 | 0.8±0.2 | 0.38 |
| **TAT – ms ( mean±SD)** | 157±16 | 140±25 | 0.17 |

**cm/s** = centimeters per second; **SD** = standard deviation; **IQR** = interquartile range; **CB** = conduction block; **cCDCB =** continuous lines of conduction delay and -block; **CV** = conduction velocity; **ECV** = electrical cardioversion; **PAC** = premature atrial complexes; **TAT** = total activation time.

**Supplemental Table 3.**

|  | **ECV**  **(N=11)** | **Control**  **(N=9)** | **P Value** |
| --- | --- | --- | --- |
| **CB**  **Prevalence – % (mean±SD )**  **Length of longest CB line – mm ( median (IQR))** | 3.2±1.7  51±24 | 3.2±2.1  40±14 | 0.95  0.29 |
| **cCDCB**  **Prevalence - % (mean±SD)**  **Length of longest cCDCB line – mm (mean±SD)** | 3.7±1.9  66±25 | 4.2±1.9  61±24 | 0.60  0.68 |
| **CV – cm/s (mean±SD)** | 87±7 | 87±7 | 0.97 |
| **Variation of CV (Δ P5-P95)** | 130±9 | 127±9 | 0.57 |
| **P_5_ of unipolar voltages – mV (median (IQR))** | 0.6 (0.5 – 0.9) | 1.0 (0.6 – 1.8) | 0.15 |
| **TAT – ms ( mean±SD)** | 159 (133 – 165) | 148 (132 – 249) | 1.00 |

**cm/s** = centimeters per second; **SD** = standard deviation; **IQR** = interquartile range; **CB** = conduction block; **cCDCB =** continuous lines of conduction delay and -block; **CV** = conduction velocity; **ECV** = electrical cardioversion; **PAC** = premature atrial complexes; **TAT** = total activation time.
